# Central Respiration and Mechanical Ventilation in the Gating of Swallow With Breathing

**DOI:** 10.3389/fphys.2018.00785

**Published:** 2018-06-25

**Authors:** Kofi-Kermit Horton, Lauren S. Segers, Sarah C. Nuding, Russell O’Connor, Pierina A. Alencar, Paul W. Davenport, Donald C. Bolser, Teresa Pitts, Bruce G. Lindsey, Kendall F. Morris, Christian Gestreau

**Affiliations:** ^1^Department of Molecular Pharmacology and Physiology, Morsani College of Medicine, University of South Florida, Tampa, FL, United States; ^2^Department of Physiological Sciences, College of Veterinary Medicine, University of Florida, Gainesville, FL, United States; ^3^Department of Neurological Surgery, Kentucky Spinal Cord Injury Research Center, University of Louisville, Louisville, KY, United States; ^4^Aix Marseille Univ, INSERM, INS, Inst Neurosci Syst, Marseille, France

**Keywords:** swallow, breathing, pulmonary afferents, central pattern generators, plasticity

## Abstract

Swallow-breathing coordination safeguards the lower airways from tracheal aspiration of bolus material as it moves through the pharynx into the esophagus. Impaired movements of the shared muscles or structures of the aerodigestive tract, or disruptions in the interaction of brainstem swallow and respiratory central pattern generators (CPGs) result in dysphagia. To maximize lower airway protection these CPGs integrate respiratory rhythm generation signals and vagal afferent feedback to synchronize swallow with breathing. Despite extensive study, the roles of central respiratory activity and vagal feedback from the lungs as key elements for effective swallow-breathing coordination remain unclear. The effect of altered timing of bronchopulmonary vagal afferent input on swallows triggered during electrical stimulation of the superior laryngeal nerves or by injection of water into the pharyngeal cavity was studied in decerebrate, paralyzed, and artificially ventilated cats. We observed two types of single swallows that produced distinct effects on central respiratory-rhythm across all conditions: post-inspiratory type swallows disrupted central-inspiratory activity without affecting expiration, whereas expiratory type swallows prolonged expiration without affecting central-inspiratory activity. Repetitive swallows observed during apnea reset the E2 phase of central respiration and produced facilitation of swallow motor output nerve burst durations. Moreover, swallow initiation was negatively modulated by vagal feedback and was reset by lung inflation. Collectively, these findings support a novel model of reciprocal inhibition between the swallow CPG and inspiratory or expiratory cells of the respiratory CPG where lung distension and phases of central respiratory activity represent a dual peripheral and central gating mechanism of swallow-breathing coordination.

## Introduction

Swallowing, a centrally-mediated sequential activation of the muscles of the tongue, larynx, pharynx and the esophagus, ensures adequate ingestion of saliva, liquids, and food. Its reflexive buccopharyngeal phase also plays a crucial role in protecting the lower airways from tracheal aspiration as the bolus moves through the pharynx into the esophagus (Bosma, 1957; Miller, 1982; Nishino, 1993; Jean, 2001). Since swallowing and respiration share muscular components and anatomic structures, notably those of the aerodigestive tract, these two vital functions must be highly coordinated. Coordination of swallow with breathing is resolved through interactions between the swallow and respiratory central pattern generators (CPGs) located in the medulla (Dick et al., 1993; Oku et al., 1994; Bianchi et al., 1995; Gestreau et al., 1996, 2000; Roda et al., 2002). Both animal and human studies have shown that swallows occur preferentially at phase-transitions of the respiratory and ventilatory cycle, i.e., inspiration to early-expiration (I-early Exp), early-expiration to late-expiration (early Exp-late Exp), and late-expiration to inspiration (late Exp-I) (Dick et al., 1993; Paydarfar and Buerkel, 1995; Ono et al., 1998). Swallows have also been reported to occur during inspiration, i.e., I-I or I-swallow, as well as to interrupt the inspiratory phase of breathing (interrupted-I swallow) (Feroah et al., 2002; Roberge et al., 2007; Wheeler Hegland et al., 2009; Bonis et al., 2011; Wheeler Hegland et al., 2011; Yagi et al., 2017). Overall, five types of swallow patterns have been proposed, including I-swallow, interrupted-I, I-Exp, early Exp-late Exp, and Exp-I, despite most single swallows occurring predominantly during the I-Exp and early Exp-late Exp phase-transitions (Martin-Harris et al., 2005). Thus, expiratory airflow generally precedes and follows swallow execution (Dick et al., 1993; Martin et al., 1994; Paydarfar et al., 1995; Feroah et al., 2002; Wheeler Hegland et al., 2009; Bonis et al., 2011). This relationship is thought to provide the maximum protection of the lower airways against pulmonary aspiration (Kijima et al., 1999; Yamamoto and Nishino, 2002; Martin-Harris et al., 2003, 2005; Pitts et al., 2013). By contrast, swallows occurring during the Exp-I transition or during inspiration present the highest risk for tracheal aspiration and are frequently encountered in patients with dysphagia (Terzi et al., 2007, 2010; Nishino, 2013).

Several studies show that central respiratory output is modified during swallow execution (Paydarfar et al., 1995; Kijima et al., 2000; Saito et al., 2002; Bonis et al., 2011). Pauses in respiration (“swallow apneas”) as well as resetting of the respiratory rhythm upon completion of swallowing likely reflect integration of peripheral inputs and central processes involved in swallow-breathing coordination. Moreover, manipulation of pulmonary stretch receptor (PSR) feedback through augmentation of lung inflation (Kijima et al., 2000), continuous positive airway pressure (Samson et al., 2005, 2008), changes in the respiratory rate (Yamamoto and Nishino, 2002), respiratory loading (Kijima et al., 1999), hypercapnic ventilation (Nishino et al., 1998) or removal of phasic lung inflation (Sai et al., 2004) affects swallow-breathing coordination and reduces the rate of reflex swallow expression, suggesting bronchopulmonary vagal involvement. During spontaneous breathing, vagal bronchopulmonary feedback is physiologically coupled (synchronous) with central inspiratory activity. In paralyzed animals, this relationship can be mostly preserved or altered using subject-triggered or mandatory mechanical ventilation of the lungs, respectively. We took advantage of these two modes of mechanical ventilation to investigate the role of isolated bronchopulmonary vagal involvement in swallow-breathing coordination.

This brief survey of the literature reveals that the peripheral and central mechanisms of swallow-breathing coordination remain poorly understood. Here, we manipulated the relationship of central inspiratory activity to mechanical lung inflation to test the hypothesis that swallow timing is coordinated by central respiratory activity (i.e., a centrally mediated “swallow gate”) as well as by PSR feedback (i.e., a peripherally mediated “swallow gate”). Our hypothesis is that swallowing is coordinated with breathing through two complementary mechanisms of inhibition (or negative modulation) of the swallow CPG originating from (1) central inspiratory cells (efferent copy of inspiratory-related activity) and (2) PSR feedback (sensory information transmitted to the brainstem during lung distension). These mechanisms protect the lower airways by preventing swallow during inspiration. Since these mechanisms rely on time-dependent operations such as gating and filtering, they can be viewed and referred to as a “swallow gate.” Also, the respiratory motor output expresses many forms of plasticity (Dick and Coles, 2000; Mitchell and Johnson, 2003; Morris et al., 2003) whereas (fictive) swallows are often considered “stereotypic events” with characteristic and consistent form (Doty, 1951, 1968; Kawasaki et al., 1964; Sumi, 1970; Miller and Loizzi, 1974; Miller, 1982). Since execution of protective reflexes such as cough and swallow also engage elements of the respiratory CPG (Gestreau et al., 1996, 2005; Shannon et al., 1998; Bolser et al., 2006), we hypothesized that the swallow CPG could also express plasticity, such as an enhancement of swallowing motor output upon sustained stimulation of afferent fibers. To test this hypothesis, long periods of electrical stimulation were applied to the superior laryngeal nerves (SLNs) to trigger repetitive swallowing, and swallowing-related nerve amplitudes and/or durations were analyzed within series.

## Materials and Methods

All experiments were conducted according to protocols approved by the University of South Florida’s Institutional Animal Care and Use Committee with strict adherence to all Association for Assessment and Accreditation of Laboratory Animal Care International (AAALAC), National Institutes of Health, and National Research Council guidelines.

### Surgical Protocol

Methods were as previously described (Morris et al., 2010; Ott et al., 2012). Briefly, data were obtained from seven adult cats (3.1–5.8 kg) of either sex that were part of a larger dataset that included recording the response of brainstem neurons during the protocols detailed below.

In two of seven animals, a bilateral thoracotomy (pneumothorax) was performed. Prior to initiating the surgical protocol, atropine (0.54 mg/kg i.m.) was injected to reduce mucus secretion in the airways. A Dexamethasone infusion (initial bolus of 2.0 mg/kg followed by 4.5 mg kg^-1^ h^-1^ i.v.) was used to minimize brain stem swelling and to prevent hypotension. Anesthesia induction and maintenance were with 5.0 and 1.0–3.0% isoflurane, respectively, mixed with medical grade air (21% O_2_-Balance N_2_-Airgas) until decerebration. Following induction, the trachea was intubated, and catheters were placed in the femoral arteries and veins for intravenous administration of drugs and fluids as well as to facilitate monitoring of arterial blood pressure. Periodically, arterial blood was collected and analyzed for PO_2_, PCO_2_, pH, and HCO_3_^-^ concentrations. Sodium bicarbonate solution (8.4%) was infused to correct metabolic acidosis as needed. Solutions of 6% Hetastarch or 5% Dextran in half-normal saline (0.45%), 0.04–0.1% dopamine, and 0.075–0.3 mg/ml phenylephrine in lactated Ringer solution were administered intravenously as needed to maintain a mean blood pressure of at least 75 mmHg. To reduce bleeding during and following the decerebration process, both external carotid arteries were ligated caudal to the lingual artery branch. We performed an occipital craniotomy, a midcollicular transection and suction decerebration (Kirsten and St. John, 1978). The brainstem was exposed, and the pia mater removed for insertion of tungsten microelectrodes for measurement of neuron extracellular potentials. Immediately prior to the transection, an infusion of the neuromuscular blocker vecuronium bromide or pancuronium bromide was given and maintained to ensure that the animals were paralyzed (initial bolus 0.1 mg/kg; continuous infusion 0.2 mg kg^-1^ h^-1^ i.v.). Following decerebration, the isoflurane concentration was gradually reduced to zero. Following completion of all experimental protocols, an overdose of Euthasol (85 mg/kg, i.v.) or sodium thiopental (20 mg/kg, i.v.) followed by potassium chloride (i.v.) was administered. Cardiac and respiratory activities were monitored until cessation.

### Nerve Isolation and Recording

The right hypoglossal (XII), left phrenic (Phr), left lumbar iliohypogastric (Lum), and right vagus (X) nerves were isolated from surrounding tissue and desheathed. In two of the cats, the right recurrent laryngeal nerve (RLN) was used instead of the X nerve. To monitor and record efferent nerve activities, the XII nerve, Lum nerve, and X/RLN nerve were placed in coiled or hooked bipolar silver electrodes, covered with a combination of mineral oil and petroleum jelly, and wrapped in parafilm. The Phr nerve was floated in place in a pool of mineral oil in a neck pocket, sectioned and recorded in coiled or hooked bipolar silver electrodes. All nerve activity was amplified, full-wave rectified, low-pass filtered, and RC integrated (τ = 200–500 ms). Integrated nerve discharge activity was used to indicate stimulus effectiveness. Integrated nerve activity, tracheal pressure (TP), end tidal CO_2_, and arterial blood pressure were monitored on a Grass polygraph and recorded digitally (16-bit, 25 kHz per channel) onto a hard disk drive for later off-line analysis.

### SLN Isolation and Stimulation

The SLNs were isolated bilaterally; each was connected to a silver bipolar electrode and covered with a combination of mineral oil and petroleum jelly or a pledget soaked in mineral oil until the nerve was used for electrical stimulation. Fictive swallowing was evoked by electrical stimulation (pulse duration, 0.25 ms; frequency, 5–20 Hz, Voltage 2.6–4.0 V, 33.3–51.5 μA, train duration 2–120 s) and identified by changes in activities of the Phr, XII, and X/RLN nerves.

### Water Bolus Stimulation

Water bolus evoked fictive swallows were elicited via rapid (less than 5 s) injection of distilled water (5–25 mL) through a polyethylene tube inserted into the mouth of each animal. A minimum of three water injection trials were performed with an inter-trial interval of at least 2 min.

### Ventilation Mode Protocols

Since we hypothesized that PSR feedback contributes to swallow-breathing coordination, we altered the timing of PSR feedback within the respiratory cycle by using two ventilation modes. In subject-triggered mode (ST), the integrated phrenic signal was used to trigger the ventilator to inflate the lungs and to allow passive deflation; in this mode, vagal feedback is mostly synchronous with central inspiration. Due to this thresholding process, there was a delay (mean duration: 458 ± 140 ms) between the end of the inspiratory phase (Phr peak) and the end of lung inflation (TP peak). In mandatory ventilation mode (MV), the ventilator rate was set to 30 breaths per minute with a gas flow rate adjusted to maintain arterial PCO_2_ at 30 ± 0.5 mmHg; in this mode, vagal feedback may be mostly asynchronous with central inspiration.

### Analyses and Statistics

Integrated activities of the Phr, X/RLN, and XII nerves were marked and coded for measurements of swallow motor output (time to peak of burst activity, total duration of burst activity, peak amplitude, area under the curve, the delay between the starts and peaks of the XII and X/RLN bursts) recorded during each stimulation-ventilation combination. The swallow duration was defined as the interval between the start of XII nerve burst activity and the end of X/RLN nerve burst activity, or the interval between the start and the end of XII nerve burst when the end of X/RLN nerve burst activity was observed before the end of XII nerve activity. The 20 respiratory and ventilatory cycles that preceded each stimulation period were marked and coded for use as control to assess any swallow related changes in respiratory and ventilatory function. The averaged durations of control respiratory cycles (T_TOT_) computed from the integrated Phrenic activity (Phr) between subject-triggered (ST) and mandatory ventilation (MV) modes were compared. In addition, the control ventilatory cycles (Vent Cycle) computed from the tracheal pressure signal (TP) between ST and MV modes were compared. To evaluate and compare the relationship between central inspiratory output and lower airway feedback during ST and MV, we calculated the overlap of the inspiratory portion of each individual respiratory cycle and coincident lung inflation(s) for both modes of ventilation. These values were expressed with regard to central inspiration or lung inflation by dividing the overlap by the duration of central inspiratory output or by the duration of coincident lung inflation(s), respectively, and compared across ventilation modes using paired *t*-tests.

To confirm which respiratory parameters changed when the mode of ventilation was switched, an initial comparison (paired *t*-tests) between control T_TOT_ and Vent Cycle measurements was performed. Differences in swallow motor output were initially compared across all stimulus ventilation conditions using a two-factor ANOVA (with Bonferroni corrections). The effect on resetting of the respiratory cycle that accommodates a swallow was analyzed using a three-factor ANOVA (with Bonferroni corrections) for single swallows. The magnitude of change in the preceding inspiratory activity of these respiratory cycles was assessed by linear regression analysis across conditions. The strength of the association between each single swallow type and the ventilation modes as well as the phase preference within the Vent Cycle for SLN-MV swallows were analyzed using a Chi Square test of independence, and a one-way repeated measures ANOVA was performed to compare the duration of swallow motor output (overall swallow duration, XII and X/RLN duration) in a series of repetitive swallows. All analyses were performed in the program SPSS Statistics version 23 (IBM, Armonk, NY, United States). Values were considered significant when *p* < 0.05.

## Results

### Patterns of Breathing and Mechanical Ventilation in Control Periods

**Table 1** compares the respiratory and ventilatory parameters of control cycles measured during the two ventilation modes. The durations of the respiratory and mechanical ventilation cycles did not differ significantly in ST and MV modes, nor did the percent duration of central inspiration associated with concurrent vagal feedback from the lungs. By contrast, the percent duration of lung inflation and vagal feedback concurrent with central inspiration was significantly lower in MV; thus, the change in ventilation mode (ST to MV) altered the relationship between vagal feedback and the respiratory cycle mainly during the expiratory phase.

**Table 1:** A comparison of control respiratory and ventilatory parameters (mean ± SD).

	ST	MV	*n*
T_TOT_ (ms)	2329 ± 590	2730 ± 927	10
Vent cycle (ms)	2319 ± 578	2046 ± 63	10
Overlap with CI (%)	69 ± 10	60 ± 11	10
Overlap with LI (%) ^∗∗∗^	60 ± 5	42 ± 9	10

^∗∗∗^*p < 0.001. T_*TOT*_ = duration of respiratory cycle; Vent Cycle = duration of ventilatory cycle; Overlap = duration of lung inflation(s) coincident with inspiratory portion of T_*TOT*_; CI = central inspiration duration; LI = lung inflation duration*.

### Patterns of Cranial Nerve Activities in Swallowing: General Features

**FIGURE 1:**
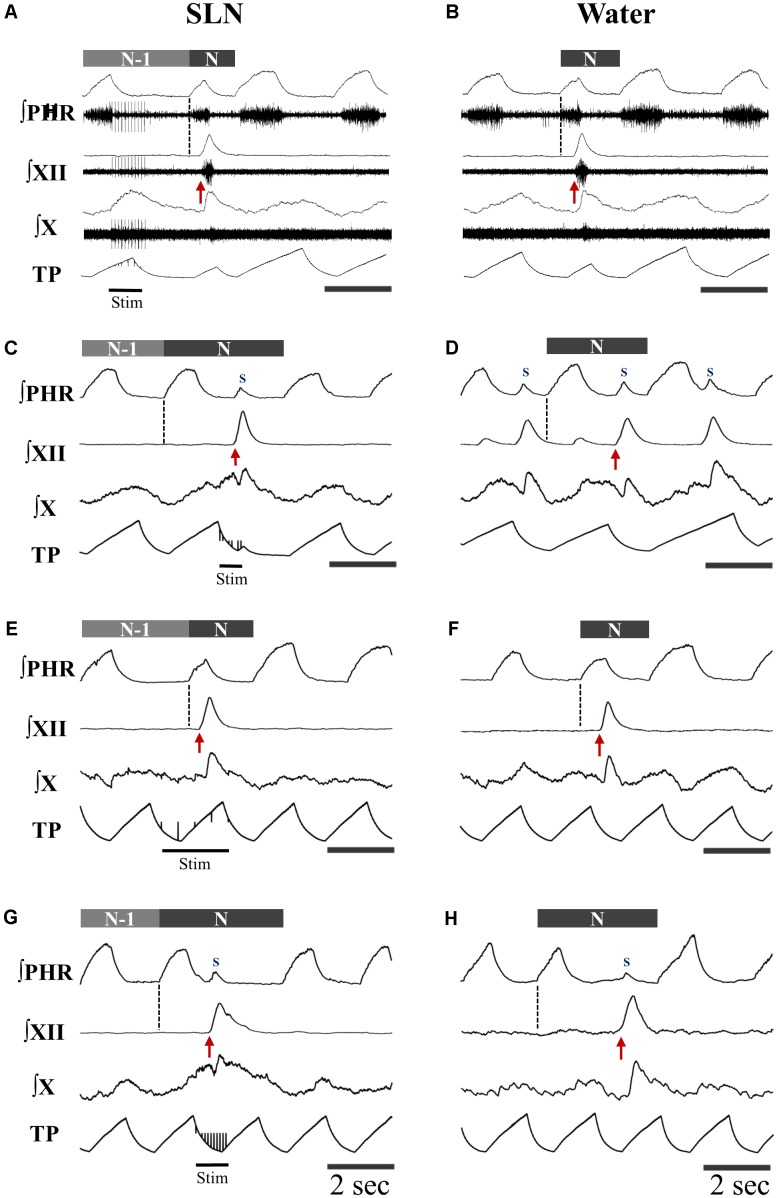
Patterns of motor activities and artificial ventilation during breathing and swallowing in the various conditions. **(A–D)** Subject-triggered (ST) ventilation. **(E–H)** Mandatory ventilation (MV). Distinct bursts on the integrated hypoglossal (XII) and vagal (X) raw nerve activities, as shown in **(A,B)**, were used to identify single swallows elicited by electrical stimulation of the superior laryngeal nerve (SLN) **(A,C,E,G)** or pharyngeal water injection **(B,D,F,H)**. Swallows were classified as post-inspiratory (Post-I) or expiratory (Exp) according to the timing of XII burst initiation (red arrow) with respect to peak phrenic activity. Post-I swallows **(A,B,E,F)** occurred after the peak of a preceding phrenic (Phr) activity and before the end of its decrementing post-inspiratory activity, whether or not the ongoing inspiratory phase was interrupted. Exp swallows **(C,D,G,H)** occurred after full completion of the Phr burst including its decrementing post-inspiratory activity. Swallows were associated with a small non-respiratory Phr burst, also known as Schluckatmung (S), which occasionally triggered the mechanical ventilator in ST mode **(C)**. When an Exp swallow occurred **(C,D,G,H)**, the S was visible and separate from inspiratory-related Phr activity (indicated by black dashed line). By contrast, when a Post-I swallow occurred, S often merged with the preceding inspiratory-related Phr activity, resulting in a small indentation on the Phr envelope **(A,B,E,F)**. Note the shorter or longer duration of the cycles which contain a Post-I or a Exp swallow (N), respectively, compared to previous control respiratory cycles (N-1).

Single or repetitive pharyngeal phases of fictive swallowing, herein referred to as swallows (*n* = 807), were evoked by electrical stimulation of the SLN or by water injection in the pharyngeal cavity (Water) in the two ventilation modes (ST and MV). The number of swallows produced by each stimulation type and in each ventilation mode are reported in **Table 2**. **Figure 1** shows representative examples of nerve recordings during breathing and swallowing in the various conditions. A variable (usually small) non-respiratory Phr burst also known as phrenic-breakthrough or Schluckatmung (S in **Figure 1**) (Jodkowski and Berger, 1988; Grelot et al., 1992; Gestreau et al., 1996) was often associated with swallow. This burst was coincident with the onset of the swallow, i.e., in phase with the swallow-related XII nerve activity. The start of the XII burst corresponded to the onset of swallowing (arrows in **Figure 1**) and always preceded the X/RLN burst. This delay between the two cranial nerves reflects the sequential motor output to tongue muscles and laryngeal adductors during swallowing. Its mean duration computed with all swallows was 134 ± 46 ms, and did not differ significantly across conditions [*F*(3,34) = 1.82, *p* = 0.16]. The mean XII and X/RLN burst durations were 771 ± 258 and 771 ± 222 ms, respectively, and the corresponding mean duration of the swallow motor sequence was 905 ± 249 ms. These parameters did not differ across conditions [*F*(3,34) = 1.35, *p* = 0.28, for XII; *F*(3,34) = 2.36, *p* = 0.09; for X/RLN; *F*(3,34) = 2.47, *p* = 0.08, for the swallow motor sequence]. Repetitive swallows within the SLN-MV condition yielded significant changes in cranial nerve burst duration (see facilitation of swallowing motor output below).

**Table 2:** The number of fictive swallows elicited during each stimulation-ventilation condition.

	SLN-ST	SLN-MV	Water-ST	Water-MV	Total
Single swallows	173	128	45	32	**378**
Repetitive swallows	57	357	9	6	**429**
Total swallows	230	485	54	38	**807**

### Swallowing and Its Relationship to the Central Respiratory Cycle Across Conditions

#### Types of Swallows and Patterns of Spinal Nerve Activities

**FIGURE 2:**
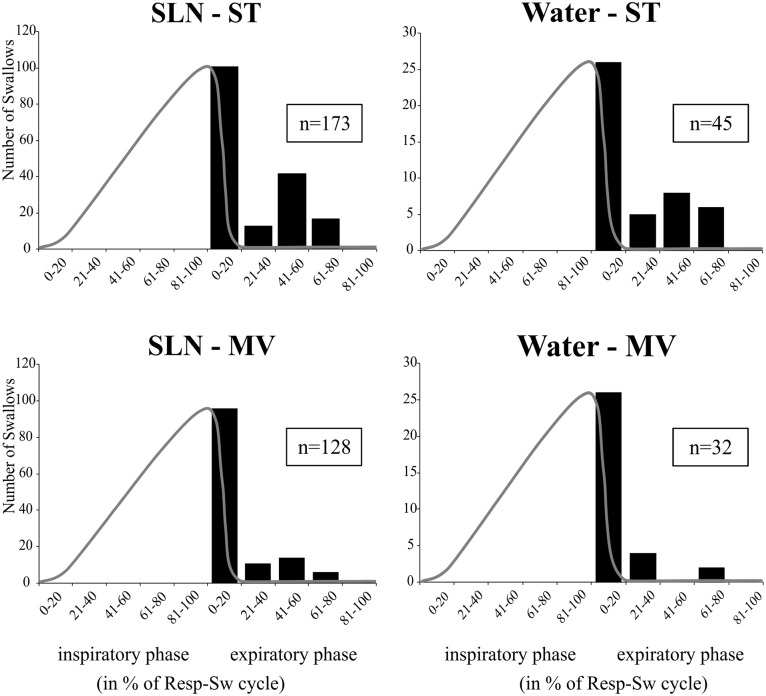
Swallowing and its relationship to the central respiratory cycle across conditions. Histograms show isolated swallow occurrences within normalized respiratory-swallow cycles, where both the inspiratory and expiratory phases are represented as percentiles. A template of a control phrenic envelope derived from averaged integrated Phr nerve signals during each stimulus-ventilation condition (gray line) has been superimposed on the plot to better represent the inspiratory portion of the central respiratory activity. Note that none of the swallows occurred in mid-inspiration and were followed by resumption of inspiratory-related Phr burst. Numbers in boxes represent numbers of single swallows analyzed per condition. SLN, electrical stimulation of the superior laryngeal nerves; Water, pharyngeal injection of water; ST, subject-triggered ventilation mode; MV, mandatory ventilation mode.

Analyses of phase-relationship between breathing and swallowing were performed using single swallows (*n* = 378) initiated within a respiratory cycle in SLN-ST, SLN-MV, Water-ST, and Water-MV (**Table 2**). Those respiratory cycles that contained a swallow were referred to as respiratory-swallow (Resp-Sw) cycles. Swallows were classified according to their timing of initiation within the Resp-Sw cycle. **Figure 2** illustrates the swallow-breathing relationship obtained with all the swallows evoked in the four conditions mentioned above. Swallow initiation in mid-inspiration that was followed by resumption of inspiratory-related Phr burst activity was never observed (**Figure 2**). Swallows were subsequently classified into two types. The post-inspiratory (Post-I) type corresponded to single swallows that initiated after the peak of a preceding Phr activity and before the end of its decrementing post-inspiratory activity, whether or not the inspiratory phase of the preceding phrenic was interrupted (**Figures 1A,B,E,F**). The expiratory (Exp) type corresponded to single swallows initiated later in the expiratory phase of breathing, after full completion of the Phr burst including its decrementing post-inspiratory activity (**Figures 1C,D,G,H**). When Exp swallows occurred, the S burst was clearly identified and distinct from the inspiratory-related Phr activity (**Figures 1C,D,G,H**). For Post-I swallows the S burst was generally merged with the preceding respiratory-related Phr activity although an additional waveform was observed after the peak Phr activity and resulted in a small indentation on the Phr envelope (**Figures 1A,B,E,F**). This swallow-related Phr activity was excluded from the computation of inspiratory-related Phr duration and amplitude. It was beyond the scope of this study to further analyze this event across conditions.

#### Changes in Swallow-Breathing Relationship

The distribution of Post-I and Exp swallow observations were combined, across stimulation types, and the total number of observations in both ventilation conditions were compared using a Chi Square Test of Independence (**Table 3**). The analysis confirmed a significant association between the increased observation of Post-I swallows and the MV ventilation mode as well as between the increased observation of Exp swallows and the ST ventilation mode. See **Table 3** for statistics.

**Table 3:** 2x2 Chi Square Test of Independence contingency table analysis of the ventilation mode preference for both swallow types observed across stimulation conditions.

	Post-inspiration		Expiration		Total Sw	
	*N*	%	*N*	%	*N*
ST	125	57	93	43	218
^∗∗∗^					
MV	121	76	39	24	160

*^∗∗∗^χ^2^(1) = 13.576, *p* ≤ 0.001*.

#### Post-I and Exp Swallows Exert Distinct Effects on Respiratory Rhythm

**FIGURE 3:**
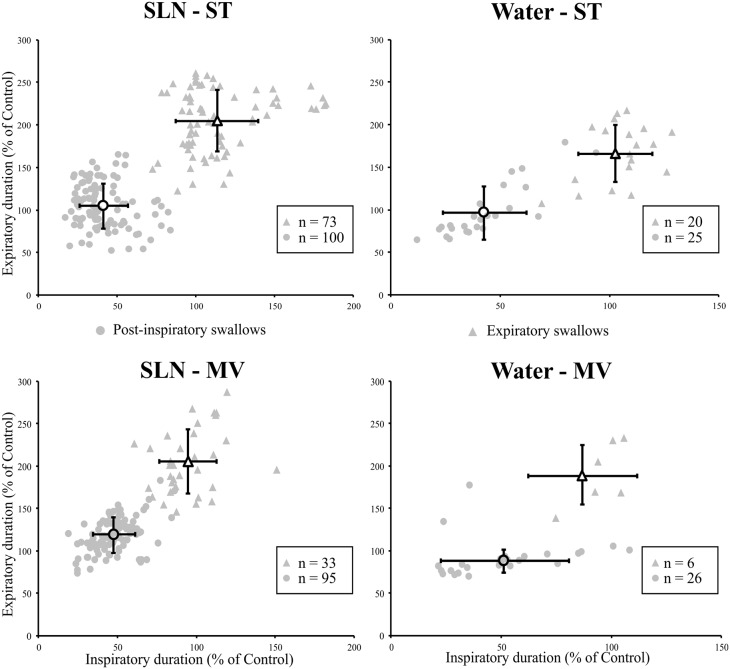
Post-inspiratory (Post-I) and expiratory (Exp) swallows exert distinct effects on respiratory-swallow (Resp-Sw) cycle duration across conditions. For each type of swallow in each ventilation and stimulation mode, both inspiratory and expiratory portions of the Resp-Sw cycle were normalized and compared to values obtained during control respiratory cycles. Note the shortening of the inspiratory phase preceding a Post-I swallow, and the lengthening of the expiratory phase associated with Exp swallows in both ventilation modes and for both types of stimuli. SLN, electrical stimulation of the superior laryngeal nerves; Water, pharyngeal injection of water; ST, subject-triggered ventilation mode; MV, mandatory ventilation mode (mean ± SD).

The effects of the two types of swallow on Resp-Sw cycles were analyzed across conditions. The durations of Resp-Sw cycles calculated in the various conditions were normalized to the average duration of corresponding control breathing cycles, in which no swallow was evoked (T_TOT_). Then normalized Resp-Sw cycle parameters were averaged per type of swallow, ventilation mode, and stimulation (**Figure 3**). Then, a three-way analysis of variance (ANOVA) was performed to compare the duration of the whole Resp-Sw Cycle, its inspiratory and expiratory portions, and the amplitude of the integrated Phr activity (Supplementary Table 1). All parameters significantly differed across conditions. There was no ventilation mode or stimulation type effect, but highly significant main swallow type effects in each of the four conditions (Supplementary Table 1). Pairwise comparisons showed that the duration of Post-I Resp-Sw cycles were significantly shorter than Exp Resp-Sw cycles. This difference was due to a significant reduction of the inspiratory phase preceding Post-I swallows (∼47% of control), and a significant prolongation of the expiratory phase (∼192% of control) associated with Exp swallows (**Figure 3** and Supplementary Table 1). In addition, the amplitude of the integrated Phr burst preceding Post-I swallow was significantly smaller (∼60% of control) than that associated with Exp swallows (Supplementary Table 1). Thus, the two types of swallows reset the respiratory rhythm by differentially affecting the inspiratory and expiratory phases of the respiratory cycle.

#### Alteration of Central Inspiratory Activity by Post-inspiratory Swallows

**FIGURE 4:**
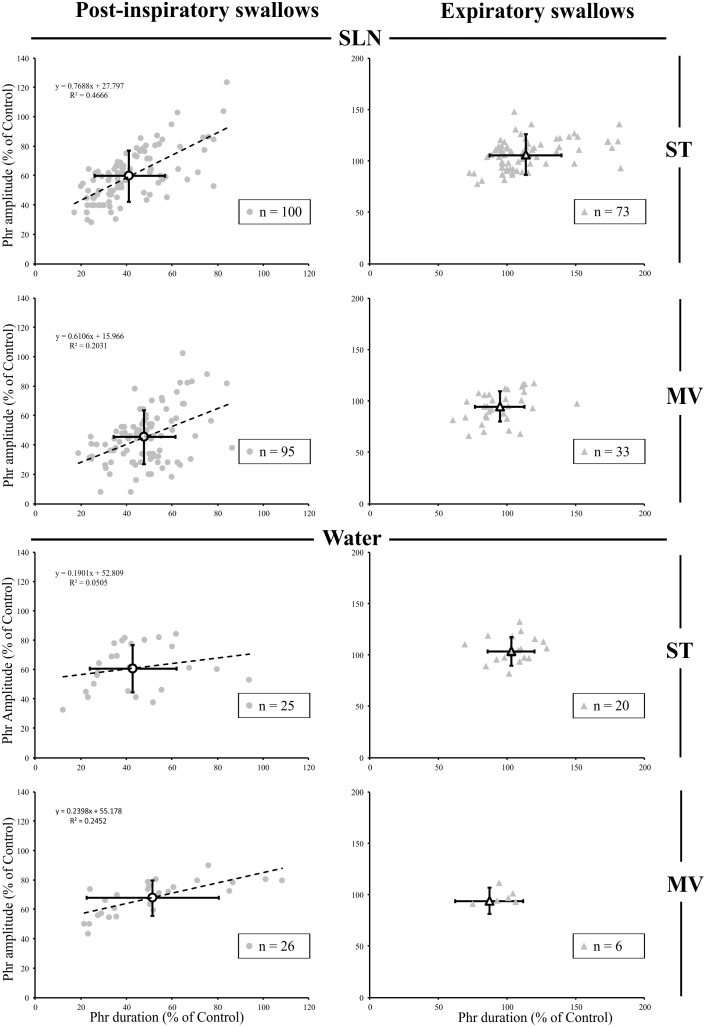
Changes in inspiratory drive preceding single swallows across conditions. For both types of stimuli (SLN and Water), and both modes of ventilation (ST or MV), the plots represent changes in duration and amplitude of the phrenic nerve (Phr) activity preceding each swallow, as measured during respiratory-swallow cycles. Phrenic parameters measured during swallow have been normalized to values measured in control breathing condition. Note the drastic reduction in inspiratory drive associated with post-inspiratory swallows (gray circles), whose average (open circles) is around 50% of control values, whereas no obvious change in this parameter was seen with expiratory swallows (averages: open triangles). Simple linear regression analyses, applied to Post-I data only, show concomitant decreases in amplitude and duration of the Phr burst preceding Post-I swallows in the SLN-ST (regression coefficient *R* = 0.77) and SLN-MV (*R* = 0.61) conditions. Note that the preceding Phr amplitude was less affected by Post-I swallows induced with water, as attested by lower regression coefficients (*R* = 0.19 and 0.24) obtained with Water-ST and Water-MV, respectively.

Since, the central inspiratory activity associated with Post-I swallows was significantly reduced (Supplementary Table 1), we further analyzed the relationship between the duration and amplitude of the preceding central inspiratory phase across conditions in individual single swallows using simple linear regression. Because the average central inspiratory duration preceding Exp swallows was similar to control (**Figure 3**) across conditions, we did not conduct a linear regression analysis on Exp swallows. In both SLN-ST and SLN-MV conditions, the results showed a range of possible inspiratory drives preceding Post-I swallows, from mostly reduced (∼20%) to almost normal (∼90%) Phr burst durations (**Figure 4**). Interestingly, there were similar changes in both the amplitude and the duration of the preceding Phr burst, as revealed by the regression equation coefficients of 0.77 and 0.61 in ST and MV, respectively (**Figure 4**). Results obtained for Water Post-I swallows in both ST and MV, also showed a variety of possible Phr durations (ranging from ∼10 to 110% of the control Phr burst duration), although the associated change in Phr amplitude was lower (regression equation coefficients of 0.19 and 0.24, respectively). Overall, we observed concomitant decreases in both the duration and amplitude of the Phr bursts that preceded Post-I swallows.

#### Respiratory Phase Resetting and Facilitation of Swallowing Motor Output During Rhythmic Swallowing

**FIGURE 5:**
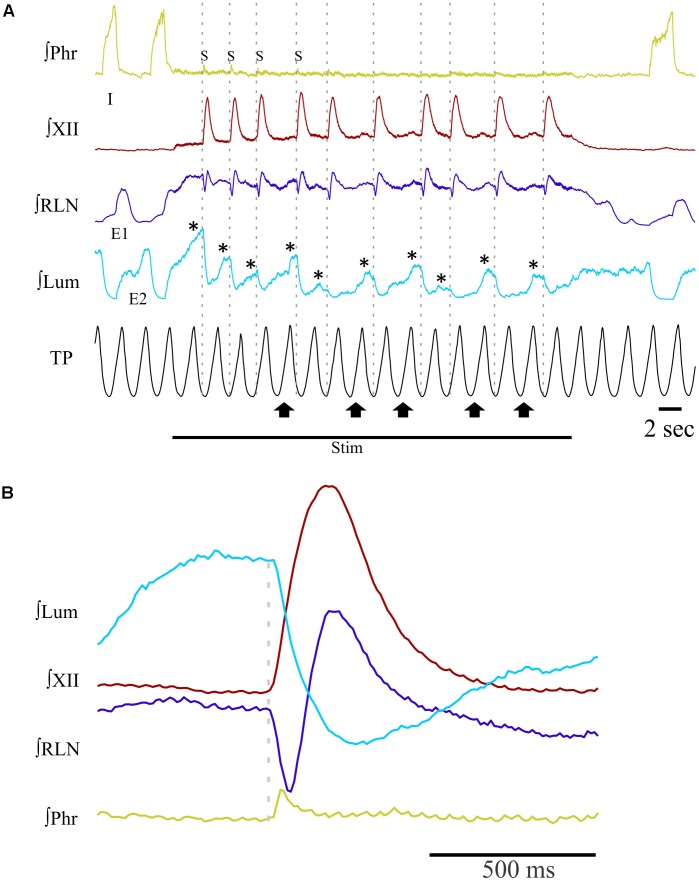
Patterns of integrated nerve activities during repetitive swallowing under apnea. Long periods (≥20 s) of SLN stimulation have been applied to trigger rhythmic swallowing and cessation of central inspiratory activity while the cats were ventilated in MV mode. **(A)** Characteristic oscillations of spinal and cranial nerves observed before, during and after rhythmic swallowing, showing rhythmic waves on recurrent laryngeal (RLN) and lumbar (Lum; stars) nerves in the absence of inspiratory-related phrenic (Phr) bursts. Note the small phrenic bursts associated with the first few swallows of the series, i.e., the so-called Schluckatmung (S). Gray dashed lines show that each swallow was induced in the lung deflation phase. Upward arrows point to inflation phases producing a shift or reset of swallow initiation to the deflation phase of the next ventilation cycle. **(B)** Averaged nerve activities during rhythmic swallow under apnea, depicting the physiological delay between the starts of the swallowing-related hypoglossal (XII) and RLN bursts. The gray dashed line corresponds to the beginning of the swallow-related XII burst. Note the increase in Lum activity before and after swallowing, whereas a decrease in Lum activity is observed during swallowing. The averaged Phr activity also displays a small burst corresponding to the S.

**FIGURE 6:**
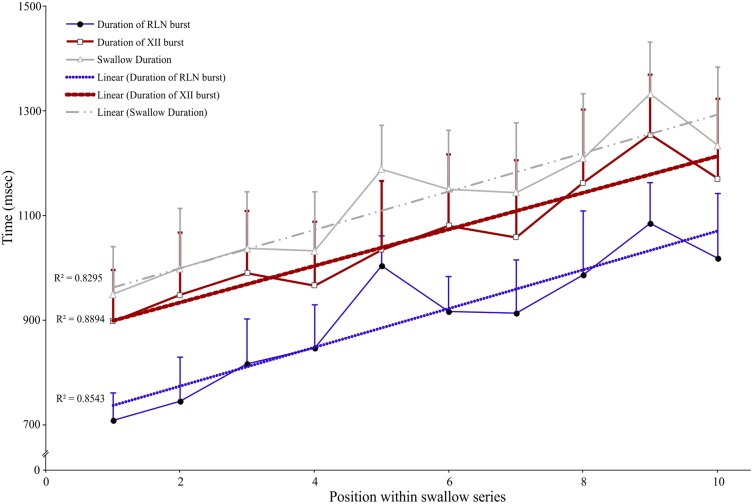
Facilitation of swallowing motor output during repetitive swallows induced by continuous SLN stimulation during apnea in MV mode. Series of 7–10 consecutive swallows were obtained, and duration of each swallowing-related burst was measured from the integrated hypoglossal (XII) and recurrent laryngeal (RLN) nerve activities and averaged. The whole swallow duration, corresponding to the time difference between the end of the RLN burst and the beginning of the XII burst or the duration of the XII burst when it persisted beyond the end of RLN burst activity, was also calculated and plotted. A one-way ANOVA with repeated measures revealed a significant main effect of the position of the swallow within a series on swallow duration [*F*(9,45) = 3.91, *p* = 0.001], mainly explained by concomitant increases in swallow-related XII and RLN burst duration (mean ± SD; *n* = 6).

Long periods (≥20 s) of SLN stimulation at 10–20 Hz were used to induce repetitive swallowing in apnea while cats were ventilated in MV mode. In these conditions, spinal and cranial nerves showed oscillations characteristic of rhythmic swallowing associated with rhythmic lumbar nerve (Lum) activity in the absence of inspiratory-related Phr bursts (stars in **Figure 5A**). To illustrate the relationship between rhythmic swallow bursts and the late-expiratory phase of breathing, swallow initiation markers (gray dashed lines in **Figure 5A**) were used as triggers to average the nerve signals (**Figure 5B**). This relationship clearly showed an increased Lum activity before and after the XII bursts, and a decrease in its activity during the swallowing-related bursts, suggesting a late expiratory (E2) phase resetting during rhythmic swallowing. In addition, a small phrenic burst associated with swallowing, i.e., a Schluckatmung (S in **Figure 5A**), was also visible as well as the delay between the starts of the XII and RLN bursts. Nerve activities from 153 of 357 repetitive apnea swallows were averaged for each animal recording and evaluated for changes in motor output throughout the first 7–10 swallows in 16 series of repetitive events. A one-way repeated measures ANOVA (**Figure 6**) revealed a significant main effect of swallow position within the swallow-series on swallow duration [*F*(9,45) = 3.91, *p* = 0.001], X/RLN burst duration [*F*(9,45) = 4.58, *p* = 0.001], and XII burst durations [*F*(9,45) = 4.44, *p* = 0.001]. The time to peak of burst activity, peak amplitude, area under the curve, and the delay between the starts and the peaks of the XII and X/RLN bursts did not differ significantly (data not shown). Thus, the observed facilitation of swallowing motor output was due to a change in duration rather than a change in amplitude of the bursts.

### Swallowing and Its Relationship to the Ventilatory Cycle

**FIGURE 7:**
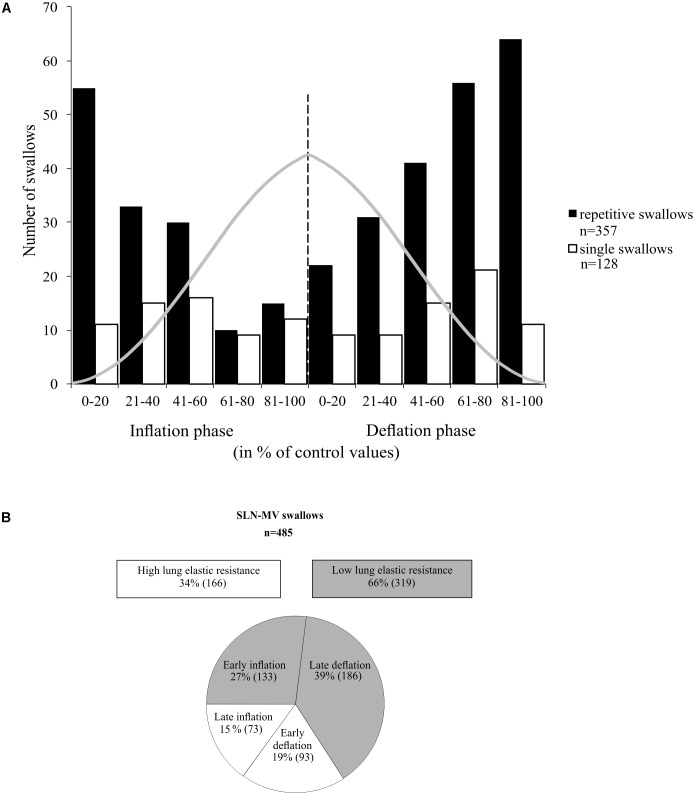
Relationship between swallowing and the ventilation cycle. **(A)** Histogram representing the distribution of single and repetitive swallows within the normalized inflation and deflation phases of the ventilation cycle. Each normalized phase is represented in percentiles (0 to 100%). Gray trace depicts tracheal pressure changes with artificial ventilation. All swallows included here, either single or repetitive, were induced by SLN stimulation and obtained in MV mode. Note that most swallows were initiated at low lung distension levels. **(B)** Measures of ventilation cycle phase preference for swallow initiation per condition. In the absence of rhythmic breathing activity, 60% of repetitive swallows were observed in the deflation phase. In addition, about 2/3 of the “inflation” and the “deflation” swallows occurred in the first and last halves of their respective phases, i.e., preferentially in early inflation or late deflation phases. See also statistical comparisons of these numbers in Section “Results.”

In many examples of repetitive swallowing evoked by sustained SLN stimulation producing apnea (MV mode) such as the one illustrated in **Figure 5A**, swallows occurred during lung deflation (gray dashed lines, **Figure 5A**). Moreover, there were lung inflation cycles that shifted swallow initiation to the deflation phase of the next Vent cycle, i.e., inducing a reset of swallowing occurrence (upward arrows, **Figure 5A**). This condition was particularly relevant to infer the proper effect of mechanical ventilation on the phase preference for initiation of swallow, i.e., without central inspiratory activity that also exerts a powerful gating *per se*, as described in previous paragraphs of results. Therefore, we plotted, and compared, the distribution of repetitive and single swallow initiations, normalized within the SLN-MV Vent cycle (**Figure 7A**). A comparison of overall swallow initiation occurrences for repetitive vs. single swallows (Supplementary Table 2) showed that when central respiratory activity is present (single swallows) both the inflation and deflation phases of the Vent Cycle are almost equally permissive to swallow initiation (49 vs. 51%, respectively). By contrast, in the absence of central respiratory activity (repetitive swallows) the inflation phase is less permissive than the deflation phase (40 vs. 60%) for swallow initiation. There was a tendency toward a deflation phase preference for swallow initiation in both types of swallow [2x2 contingency table analysis; χ^2^(1) = 3.24, *p* = 0.072]. The histogram (**Figure 7A**) shows, in both repetitive and single swallows, fewer swallows initiating later in the inflation phase and early in the deflation phase of the Vent Cycle. To better understand this difference, the inflation and deflation phases were divided into Early-half and Late-half phases and the number of initiations within each half phase was compared separately for each condition in a 2x2 contingency table. The analysis confirmed that fewer swallows were initiated (Supplementary Table 2) from the Late-half phase of inflation through the Early-half phase of deflation, in both single [χ^2^(1) = 5.25, *p* = 0.022] and repetitive [χ^2^(1) = 44.15, *p* < 0.001] swallows. The observed negative modulation effect was more strongly associated with repetitive swallows (φ_c_ = 0.352) than single swallows (φ_c_ = 0.203). Collectively, these data showed that vagal afferent activity associated with mechanical inflation of the lungs altered the timing of swallow initiation, suggesting a peripheral gating.

## Discussion

In this study we made an in-depth investigation of swallow-breathing coordination using various stimulus-ventilation conditions. Our results identified two types of single swallows producing distinct effects on central respiratory activity. Also, repetitive swallows during apnea reset the late expiratory phase of breathing and are associated with facilitation of swallow motor output. In addition, we provided evidence for a negative modulation of swallow initiation by vagal feedback, including a resetting by lung inflation (peripheral gating). These findings allowed us to propose a conceptual framework and infer the potential connectivity between brainstem CPGs responsible for swallow-breathing coordination.

### Methodological Considerations

This study was performed in decerebrate, paralyzed and artificially ventilated animals. Therefore, it concerns reflex-fictive (but not volitional) swallowing, without functional feedback in response to activation of muscles, and in the absence of supra-mesencephalic structures. This model allowed us to focus on brainstem mechanisms involved in swallow-breathing coordination. Both electrical stimulation of SLN and pharyngeal water injection were used as stimuli to trigger swallow and compare their effects on breathing pattern. As these two distinct stimuli activate different afferent pathways, we hypothesized that common effects may be due to shared central mechanisms that govern swallow-breathing coordination, while different results may be due to changes in peripheral inputs. Also, two different ventilation modes were used to alter the physiological synchrony between central inspiratory activity and lung inflation, and to study the effect of volume-related vagal feedback on swallowing. Finally, the electrophysiological analyses reported here were performed at the motor output level as a first step to decipher the precise time relationships between breathing and swallowing.

### Swallow Types and Their Relationship With Central Respiratory Activity

As pointed out in the introduction, single swallows can occur preferentially at phase-transitions of the respiratory cycle, and five swallow patterns have been proposed in the literature (Dick et al., 1993; Martin et al., 1994; Paydarfar et al., 1995; Ono et al., 1998; Feroah et al., 2002; Martin-Harris et al., 2005; Wheeler Hegland et al., 2009; Bonis et al., 2011). Here, we showed that single swallows could be divided in two types based on their timing of occurrence within the swallow-breathing cycle, and their distinct effects on central respiratory activity. Post-I swallows were tightly coupled to, and mainly interfered with, the preceding inspiratory phase of breathing, whereas Exp swallows occurred later in expiration, did not influence inspiration, and prolonged the expiratory phase of breathing. Since both stimuli used to trigger swallow resulted in similar breathing alterations, the two distinct relationships identified herein may reflect common central (brainstem) processes engaged in swallow-breathing coordination. These differential effects of swallowing on breathing pattern have been consistently reported in both animal and human studies (Nishino et al., 1985; Terzi et al., 2010; Bautista et al., 2014), but the underlying mechanisms are unknown. Of note, swallows that start and finish during inspiration (I-swallow) or swallows followed immediately by inspiration (Exp-I swallow) were not observed in our study. This discrepancy may be due to differences in experimental protocols or interspecies differences, as goats, sheep, or lambs but not cats can swallow efficiently in inspiration (Feroah et al., 2002; Roberge et al., 2007; Bonis et al., 2011). Alternatively, we believe that the lack of I-swallows and Exp-I swallows indicate that the inspiratory phase and the expiratory to inspiratory phase-transition are poorly permissive to trigger a swallow in cats. Also, the Exp-I swallow type reflect a disruption of the normal temporal coordination between swallow and breathing, increasing the risk of aspiration, as suggested earlier (Terzi et al., 2010). Interestingly, the way Post-I swallows altered the preceding inspiratory drive in the present study suggests that swallow can interrupt ongoing inspiratory activity at any time. Earlier occurrences of swallow initiation resulted in premature termination of central inspiration, while later swallow initiation was associated with a roughly normal inspiratory drive. Thus, “interrupted-I” type of swallow may not be different from those Post-I swallows occurring earlier during the inspiratory phase of breathing. By contrast, it is widely accepted that Exp swallows result in a reflex prolongation of expiration, thereby decreasing the risk of tracheal aspiration (Paydarfar et al., 1995; Wheeler Hegland et al., 2009; Terzi et al., 2010; Bautista et al., 2014). Overall, the differential effects of Post-I and Exp swallows on breathing seem to be related to a dynamic processing of swallow-related signals which is time- and activity-dependent. This central processing may be viewed as a “central swallow gate.” Brainstem respiratory oscillations may turn on and off functional connectivity between the swallow central pattern generator (swCPG) and inspiratory or expiratory elements of the respiratory central pattern generator (rCPG), as suggested by modeling studies supporting dynamic routing of information (Akam and Kullmann, 2010). Swallow-breathing patterns observed during repetitive swallows in the absence of inspiratory activity, notably alternate E2 and swallow oscillations, provide additional support to an interaction between swCPG neurons and expiratory cells. Further studies are needed to better understand these dynamic processes.

### Facilitation of Swallowing Motor Output During Rhythmic Swallowing

Our results show an increase in duration, but not amplitude, of cranial nerve bursts within the swallow-series associated with SLN-induced repetitive swallowing during central apnea. This change in swallowing motor output suggests that the swCPG is subjected to short-term plasticity (Atwood and Karunanithi, 2002; Pan and Zucker, 2009). This observation is in marked contrast with the classical view that considers fictive swallows as stereotypic events with characteristic and consistent form (Doty, 1951, 1968; Kawasaki et al., 1964; Sumi, 1970; Miller and Loizzi, 1974; Miller, 1982; Dick et al., 1993). At the synaptic level, facilitation, augmentation and potentiation are various forms of short-term plasticity that can be observed after few seconds, 10s of seconds or several minutes of stimulation, respectively (Zucker, 1999). These forms of activity-dependent plasticity affect the probability of transmitter release or the amount of transmitter that is released and are attributed to presynaptic mechanisms such as an elevation of the residual intracellular calcium concentration. Here, the increase in swallowing motor output was observed after a few seconds of SLN stimulation and is thus referred to as facilitation. Interestingly, repetitive stimulation of the SLN *in vivo* or of the solitary tract *in vitro* has been shown to trigger augmentation and short-term potentiation of hypoglossal inspiratory discharges or potentiation of post-synaptic potentials, respectively (Fortin et al., 1992; Budzinska and Wojtal, 2001). Further, activation of *N*-methyl-D-aspartate receptors within the NTS mediates a respiratory short-term potentiation *in vivo* (England et al., 1992). Since SLN fibers run in the solitary tract and release glutamate in NTS to trigger swallowing (Jean, 2001), facilitation of cranial motor output during swallowing may occur in NTS. Cellular mechanisms of facilitation within the NTS may involve activation of presynaptic nicotinic cholinergic receptors (Kalappa et al., 2011). Facilitation could also be mediated by serotonin as focal application of serotonergic agonists in the NTS was shown to selectively facilitate glutamate-evoked pharyngeal stage of swallowing (Bieger, 1981; Hashim and Bieger, 1987). Lastly, the expression of swallow facilitation may require activation of endocannabinoid mediated retrograde signaling within the NTS (Mostafeezur et al., 2012).

### Evidence for a Peripherally-Mediated Swallow Gate

During breathing, PSRs afferents convey lung volume related information via the vagus nerve to NTS pump cells (Berger and Dick, 1987; Davies et al., 1987; Bonham and McCrimmon, 1990; Kubin et al., 2006). This information is further relayed to the lateral pons and the ventral respiratory column to finely tune central respiratory activity through the Hering–Breuer reflex (HBR) (Davies et al., 1987; Ezure and Tanaka, 1996; Ezure et al., 1998, 2002; Kubin et al., 2006). The majority of swallow-breathing coordination work in animals has focused almost exclusively on the preferred respiratory phase for swallow initiation (Dick et al., 1993; Gestreau et al., 1996; Ono et al., 1998; Feroah et al., 2002; Bonis et al., 2011, 2013). However, significant work in humans has defined a relatively small lung volume (∼45–65% vital capacity) in which swallow occurs regardless of respiratory phase (Wheeler Hegland et al., 2009, 2011; Martin-Harris and McFarland, 2013). While we agree there is considerable evidence for an expiratory phase preference during normal swallow events, we hypothesize that this may be due in large part to predictable lung volumes. Additionally, conditions which shift tidal volume [i.e., chronic obstructive pulmonary disease (COPD) (Gross et al., 2009) or laparotomy (Pitts et al., 2015)]; and/or alter vagal feedback [head and neck cancer (Martin-Harris et al., 2015)] shift swallow preference to inspiration leading to increased aspiration risk. We therefore suggest that there is an inhibitory lung-volume reflex mechanism mediated by PSRs that reduces swallow initiation from the late inflation phase through the early deflation phase. This result is consistent with most swallow apneas being associated with low elastic lung resistance, i.e., late in the expiratory or early in the inspiratory phase of the ventilation cycle (Costa and Lemme, 2010). Moreover, the shapes of the distribution between single and repetitive swallows (**Figure 7A**) show that as lung elastic resistance increases, the lungs function more efficiently to negatively modulate swallow initiation in the absence of the “central swallow gate.” Our observations also support the view that mechanical feedback provides afferent surveillance information, required by the rCPG to allow swallow execution at preset lung volumes, that facilitates gas exchange during the swallow apnea (Paydarfar, 2011). In the absence of rCPG activity, i.e., during apnea, the mechanical feedback seems sufficient to supplant the “central swallow gate.” Such afferent surveillance information may regulate swallow initiation and coordination in order to maintain a reserve lung volume that can be used in the execution of other airway defense behaviors such as cough (Pitts et al., 2013). In this view, the NTS may play a crucial role. Indeed, this nucleus processes lower and upper airway inputs, is thought to contain interneurons forming the swCPG (Jean, 2001) in addition to the dorsal respiratory group neurons (Gestreau et al., 1996), and has been proposed to orchestrate airway protective behaviors such as cough and swallow (Troche et al., 2014; Bolser et al., 2015).

### Conceptual Framework and Inferred Brainstem Neuronal Connectivity

**FIGURE 8:**
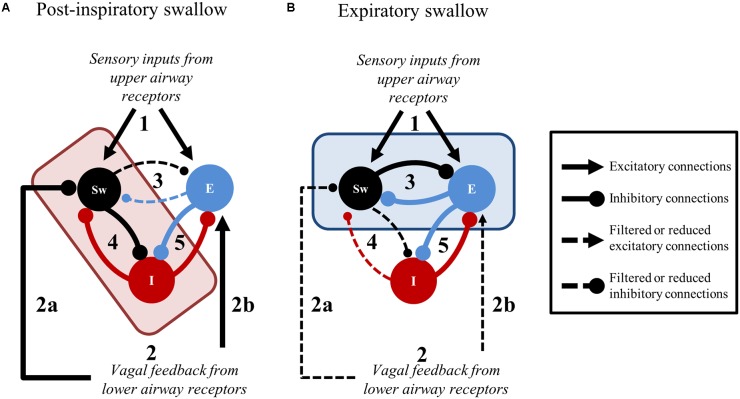
Suggested central and peripheral connectivity for swallow-breathing coordination. In both panels, stimulation originating from upper airway receptors (1) and feedback from the lower airways (2) is relayed to swallow (Sw) and expiratory (E) neurons. **(A)** Post-inspiratory swallows result when the medullary network receives convergent upper and lower airway information. The convergence of 1 and 2 in the brainstem shifts the connectivity of the central gate (red rectangle) to filter signaling between Sw and E neurons (3) in favor of Sw and inspiratory (I) neuron connectivity (4). The peripheral gate inhibits Sw neurons (2a) until the excitation of E neurons (2b), that subsequently inhibit I neurons (5), indirectly dis-inhibits Sw neurons through cessation of inspiration. Consequently, Sw neuron inhibition on I neurons increases (4) to strengthen the termination of inspiration. **(B)** Expiratory swallows result when upper airway information is received by the medullary network in the presence of decrementing lower airway feedback. When peripheral gating activity is decrementing as 1 is received in the brainstem, the production of swallow is driven by the connectivity shift of the central gate (blue rectangle) that filters signaling between Sw and inspiratory (I) neurons (4) in favor of Sw and E neuron connectivity (3). Line thickness represents the relative weight of these suggested connections.

The present findings and the existing literature support a novel framework to further scrutinize peripheral and central brainstem mechanisms involved in swallow-breathing coordination (**Figure 8**). The elegant work by Sun et al. (2011) shows that central apnea induced by SLN stimulation can be selectively abolished by pharmacological blockade of the Bötzinger Complex region, while repetitive swallows are still present and, importantly, stay coordinated with breathing. The persistence of coordination in their experiments supports our notion of a “central swallow gate” and supposes that this central gate is partly independent of the Bötzinger Complex. Also, peripheral SLN inputs directly excite swCPG neurons and indirectly inhibit central inspiratory activity (**Figure 8**). The indirect inhibition of inspiration by SLN likely depends on post-inspiratory neurons (**Figure 8A**, “E” population) of the ventral respiratory column (Sun et al., 2011), similarly to the HBR (Hayashi et al., 1996). This may suggest that peripheral inhibition of inspiration from upper and lower airways share the same neuronal substrate (**Figures 8A,B**, black lines with arrowheads converging on “E” populations). In addition, volume-related information mediated by vagal feedback would contribute to directly (**Figure 8A**) or indirectly inhibit swCPG neurons, as shown in this study (**Figures 7**, **8B**). At the central level, swallow-breathing patterns observed with Post-I or Exp swallows are consistent with a reciprocal inhibition between swCPG neurons and inspiratory or expiratory cells, respectively (**Figures 8A,B**). Since brainstem neurons were also recorded using multi-electrode arrays in these experiments, further analysis of these neuronal responses is needed to investigate the functional connectivity between swallow and respiratory neurons.

## Conclusion

This study provides evidence for two main types of swallow-breathing relationships with differential effects on central respiratory activity. We suggest that dynamic processes acting as a “central swallow gate” may operate in brainstem networks to link swCPG neurons with either inspiratory or expiratory elements of the rCPG. Processing of swallow-related inputs also depends on pulmonary feedback, which acts as a peripheral gate controlling swallow initiation. Finally, we show a facilitation of swallowing motor output, suggesting that CPGs for breathing and swallowing share many mechanisms including short-term plasticity (Hayashi et al., 2003). This phenomenon could be of interest for non-surgical and non-pharmacological rehabilitation for patients with dysphagia.

## Author Contributions

K-KH, PD, BL, KM, and CG conceived and designed the study. K-KH, LS, SN, BL, KM, PA, DB, TP, and CG designed and performed the animal experiments. K-KH, LS, SN, RO, PA, and CG analyzed the data. K-KH, LS, and CG interpreted the results obtained. K-KH, LS, SN, and CG drafted the manuscript. K-KH, LS, SN, BL, PD, KM, DB, TP, and CG revised the manuscript. All authors read and approved the final version of the manuscript.

## Conflict of Interest Statement

The authors declare that the research was conducted in the absence of any commercial or financial relationships that could be construed as a potential conflict of interest.
